# Variability in feeding of *Gammarus pulex*: moving towards a more standardised feeding assay

**DOI:** 10.1186/s12302-014-0015-4

**Published:** 2014-06-24

**Authors:** Annika Agatz, Colin D Brown

**Affiliations:** 1 Environment Department, University of York, Heslington, York YO10 5DD UK; 2 Food and Environment Research Agency, Sand Hutton, York YO 41 1LZ UK

**Keywords:** Parasite infection, Food source, C-N ratio, Toxicity testing

## Abstract

**Background:**

Focusing on feeding as an endpoint in ecotoxicological studies is a useful and sensitive tool to detect sub-lethal impacts on individual organisms with relevance to higher levels of organisation (i.e. population and ecosystem levels). We conducted a series of experiments to identify and quantify the influence of parasite infection and food source, food quality, body size and acclimation time prior to testing on the feeding rate of individual *Gammarus pulex*. Our aim was to assess the variability in feeding rate associated with these factors to support design of feeding assays with individual organisms at a daily resolution.

**Results:**

Overall, feeding rates varied enormously across experiments, and all factors were confirmed to have a significant impact on feeding rates. Reducing the intra-specific variability by using a particular sub-group within each tested factor (except acclimation time) was found to be indispensable for a successful feeding assay with individual organisms. Focusing on organisms of a sub-group in terms of parasite infection and body mass resulted in a reduction in intra-specific variability of up to 50% and 57%, respectively. Using a food source of particular quality reduced the variability by up to 38%.

**Conclusions:**

We presented a list of factors that naturally have an impact on feeding rates of *Gammarus*, quantified their impact on the variability in feeding rates, discussed their importance for consideration when planning a feeding assay and suggested some additional measurements alongside the feeding assay to improve data comparison between studies.

## Background

Focusing on feeding as an endpoint in ecotoxicological studies is a useful and sensitive tool to detect sub-lethal impacts on individual organisms with relevance to higher levels of organisation (i.e. population and ecosystem levels). Energy availability depends on feeding [1,2], and the energy budget can be considered an indicator of the overall condition of an organism [3]. Feeding determines the health of a population because altered growth and reproduction can be instigated by an effect on feeding [4–7]. Furthermore, reduced feeding can, at weak levels, reduce the possibility of survival due to interference with further sub-lethal effects and, at strong levels, cause death. Beyond secondary impacts of effects on feeding at the population level, feeding activity can play a direct role at the ecosystem level due to its importance for nutrient cycling.

One group of organisms responsible for a high proportion of the nutrient cycling in freshwaters are detritivores like *Gammarus* due to their key role for litter breakdown through fragmentation of leaf material [8,9]. Feeding of gammarids can be affected at low levels of pollution [10–14], impacts are almost instantaneous, and ex situ feeding assays are representative of leaf decomposition in the field [15]. Therefore, ex situ feeding assays are a useful tool to detect impacts with strong relevance to the ecosystem level.

Laboratory feeding assays with gammarids have been conducted for several decades [10,14,16] and follow the method of either time-response feeding experiments [17–20] or mass feeding assays [21,22]. Ecotoxicological studies have been carried out, measuring the composite feeding rate over periods from 4 to 7 days [16], and recovery potential was mostly not included (for exceptions, see [23,24]). These kinds of studies do not represent the majority of exposures to contaminants in the field. More environmentally realistic exposure regimes (short-term and fluctuating exposures) are scarce amongst communicated studies. In order to test more environmentally realistic exposures, multiple measures for the same test organisms are needed. This means that mass feeding assays are the only suitable method, because changing the food source (as is done in time-response feeding experiments) can alter the feeding rate itself. A reduction of the temporal resolution in feeding assays is likely to improve the representation of exposure in the field, but it is likely to be accompanied by increased variability in results. This arises because both the methodological uncertainty and the relative importance of intra-specific variability between test organisms will increase.

Intra-specific variability is an important influence in ecotoxicology, particularly when studying sub-lethal effects. There may be differences in the endpoint of interest amongst individuals which are so large that it may be more appropriate to focus on a sub-group of the test species in order to detect changes caused by the tested stressor. Thus, reducing the intra-specific variability in the test system will increase the statistical power to detect effects. That is the reason why such tests are mainly conducted with more than one individual per replicate, because intra-specific variability has less impact on the results using this method. The intra-specific variability in feeding rate needs to be quantified to identify which sub-group of *Gammarus* to use in feeding assays at a daily resolution.

Several natural factors are known to influence the feeding rate of organisms, such as food source, food quality, body size, temperature, reproductive status, parasite infection and water quality. For gammarids, many of these influences have been reported in the past (summarised by Kunz et al. [16]), but the quantitative influence on feeding was either not reported or only given for the food source used in the particular test without further information on the nutritional status of the food source used. Thus, identification of the quantitative impact of natural factors on feeding rate by comparison of control treatments from earlier studies is impossible. We conducted a series of experiments to identify and quantify the influence of parasite infection and food source, food quality, body size and acclimation time on the feeding rate of *Gammarus pulex*. The aim was to identify a more standardised way to measure the influence of xenobiotics on the individual feeding rate of gammarids at a daily resolution to allow the investigation of effects from short-term and/or fluctuating exposures.

## Results and discussion

For all experiments, the pH ranged between 7.4 and 7.9; the oxygen content was always higher than 75% saturation; and the temperature ranged between 12.2°C and 14.0°C. The measured pH was close to the optimum (7.2 to 7.8) for gammarids given by Schellenberg [25]. Oxygen content and temperature of the test medium fulfilled the conditions preferred by *G. pulex* [26].

### General findings

**Figure 1:**
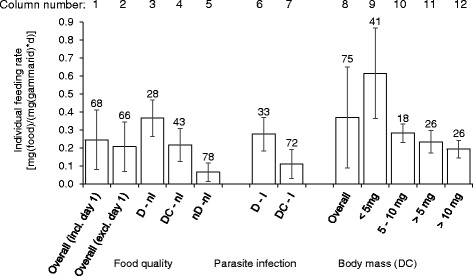
**Variation in feeding rate of individual** ***Gammarus pulex*** **.** The feeding rate was compared with type of food, presence (I) or absence (nI) of infection with acanthocephalan parasites and body mass. The food source is indicated with DC for horse chestnut leaf discs decomposed with *Cladosporium* sp., D for leaf discs decomposed in water and nD for non-decomposed leaf discs. The numbers represent the standard deviation as a percentage of the average (i.e. coefficient of variation × 100).

The overall feeding rate of the first experiment (*impact of food source and parasite infection*) was 0.21 ± 0.14 mg (food)/(mg (gammarid) × d) without distinguishing the influence of food source and parasite infection (Figure 1, column 2). This overall feeding rate ranged between 0.17 ± 0.13 and 0.37 ± 0.20 mg (food)/(mg (gammarid) × d) when calculated on a daily basis. A reduction of the variability of the test results by 1.6% was observed on discarding the first feeding period from the data analysis (Figure 1, columns 1 and 2). Data for the first feeding period (*t*_0h_ to *t*_24h_) were excluded from further analysis because of a significant difference in feeding rate relative to subsequent periods.

The overall feeding rate in the next experiment (*impact of body mass*) without distinguishing on body mass was 0.37 ± 0.28 mg (food)/(mg (gammarid) × d) (Figure 1, column 8) and ranged between 0.23 ± 0.25 and 0.51 ± 0.54 mg (food)/(mg (gammarid) × d) when calculated on a daily basis.

Overall, the intra-specific variability in feeding rate was very high. The standard deviations of the measured feeding rates were 66% and 75% of the average values for the two experiments described above when no differentiation in the three tested factors (food source, parasite infection and body mass) was made (Figure 1, columns 1 and 8). The large variability indicated a low statistical power in any tests of individual feeding rate of *G. pulex* at a daily resolution and thus the need for further work to understand and reduce intra-specific variability.

### Food source

The feeding rate of non-infected gammarids was significantly influenced (*p* < 0.001, power = 1, overall difference amongst treatment groups; ANOVA, Holm-Sidak test) by leaf type (Figure 1, columns 3 to 5) for experiments where gammarids were fed with different sources of horse chestnut. The food source nD (not decomposed) gave the lowest feeding rate (0.07 ± 0.05 mg (food)/(mg (gammarid) × d)), the feeding rate of organisms fed with source DC (decomposed with *Cladosporium* for 2 weeks) was intermediate (0.22 ± 0.09 mg (food)/(mg (gammarid) × d))), and food source D (decomposed with *Cladosporium* for 3 months) resulted in the highest feeding rate (0.36 ± 0.10 mg (food)/(mg (gammarid) × d))). Dangles and Guerold [27] found the same relationship for the freshwater amphipod *Gammarus fossarum*, and Graça et al. [28] observed that *G. pulex* ate twice as much when leaf material was conditioned*.* Other references show that food preferences for freshwater detritivores are related to the time of inoculation with microorganisms [29,30].

Results demonstrated that standardised food preparation and storage can reduce the variability of the feeding rate of *G. pulex* in laboratory studies (Figure 1, food quality) which would increase the potential for detecting stressor-related effects ex situ. Within this experiment, a maximal reduction of the variability in feeding rate by 38% was found when data were distinguished by food quality. The higher the food quality and thus the feeding rate, the lower the variability (Figure 1, food quality).

### Parasite infection

Organisms infected with acanthocephalan parasites showed a lower feeding rate for both food types tested (*p* = 0.064 and *p* = 0.099 for food sources DC and D, respectively; power = 0.999; ANOVA, Holm-Sidak test) (Figure 1). The feeding rate decreased with parasite infection from 0.36 ± 0.10 to 0.28 ± 0.09 mg (food)/(mg (gammarid) × d) when fed with food of source D, and from 0.22 ± 0.09 to 0.11 ± 0.08 (mg (food)/(mg (gammarid) × d)) by feeding with leaves of source DC (Figure 1, column 4 vs. 7). The results also suggest that the intensity of the influence may have been related to the food source. A reduction in feeding rate caused by parasite infection of 22% and 50% was found when fed with the leaf types D and DC, respectively. Excluding infected organisms from laboratory studies reduced the variability of the test results by up to 33% (Figure 1, column 2 vs. 6).

The results of the present study combined with those of Brown and Pascoe [21] show that a separation of the organisms according to whether or not they are infected with acanthocephalan parasites will reduce the variability of the test results and thus increase the power in a toxicity study to detect any effects caused by a stressor. Standardisation of either parasite infection or food quality might be suitable to reduce the intra-specific variability in the individual feeding rate for successful toxicity studies at a daily resolution. However, it might be advisable to standardise both because significant differences in feeding rate were observed for the tested food sources and infection status.

### Body size

**Figure 2:**
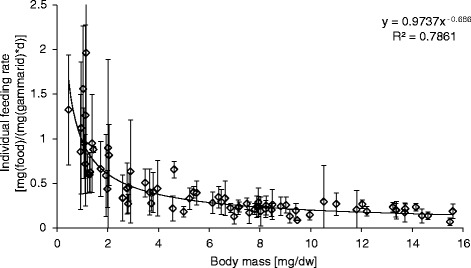
**Feeding rate of individual** ***Gammarus pulex*** **as a function of body mass.** Average ± standard deviation for a measurement period of 9 days after changing the food source from non-inoculated (nD) to inoculated food (DC).

A strong relationship (*R*^2^ = 0.79) between feeding rate and body mass (given in dry weight (dw)) was observed for all observation periods. Figure 2 shows the average of the feeding rate as a function of body mass for the whole experimental duration. The feeding rate was consistently higher for smaller organisms.

**Figure 3:**
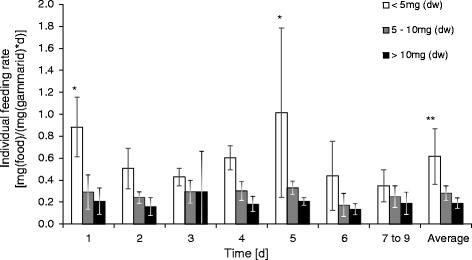
**Feeding rate of three different size classes of** ***Gammarus pulex*** **over time.** The feeding rates were taken after changing the food source from non-inoculated (nD) to inoculated food (DC). Average values (*n* > 18 for organisms <5 mg, *n* > 29 for organisms of 5 to 10 mg, *n* > 15 for organisms <10 mg). *Significant difference compared to the same group over time (*p* < 0.05, Kruskal-Wallis followed by Dunn's test); **significant difference between large and small organisms (*p* < 0.01).

Figure 3 shows the individual feeding rate over time for three sizes classes of the organism. The feeding rate of each group was compared over time, and it was found that the variability was the greatest and significant for small organisms (<5 mg), whereas for larger organisms, no significant differences in feeding rate over time were observed. Smaller organisms (body mass <5 mg) had two to three times higher feeding rates than organisms with a body mass of >5 mg when calculated over the whole experimental duration (Figure 1). Specification in terms of body mass reduced the variability in feeding rate compared to the mixed groups by 35%, 57% and 49% for the groups <5 mg, 5 to 10 mg and >10 mg, respectively.

When making feeding activity assays with *G. pulex*, it is advisable to use organisms of a very specific body mass (for example, 2.0 to 2.5 mg) to reduce the variability of the test results and thus increase the possibility of a successful toxicity study. However, restricting the size range of organisms reduces the relevance of test results for the mixed populations found in the environment. A further option would be to use organisms of a higher body mass because of the decreasing strength of the relationship between feeding rate and body mass with increasing body weight. It was observed that organisms >5 mg showed a less distinct and not significant fluctuation in feeding rate over time (*p* = 0.304 and *p* = 0.554 for organisms between 5 and 10 mg and organisms >10 mg, respectively; Kruskal-Wallis test) when compared to smaller organisms (Figure 3); however, this was offset by the disadvantage that the feeding rate was lower than that for smaller organisms (Figure 1). It was observed that the feeding rate of organisms <5 mg fluctuated greatly over time, yielding significant differences between different observation periods. Such fluctuations must be excluded for toxicity studies. Thus, the results would suggest the use of organisms >5 mg for toxicity tests. Use of larger organisms reduces uncertainties from weighing, and such organisms can be collected throughout the year [31]. We suggest the use of body length as a measure of an organism's dry weight for practicality in future feeding assays. A correlation between body length and dry weight suitable for organisms between 2 and 16 mm is given by Graça et al. [28].

### Food quality (C-N ratio)

**Figure 4:**
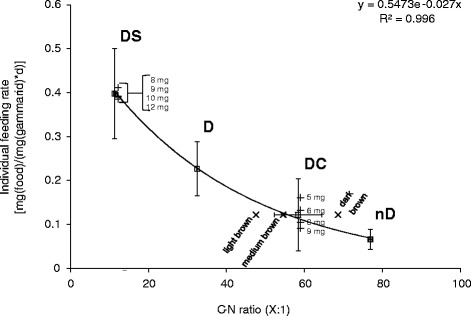
**Variation in feeding rate of individual** ***Gammarus pulex*** **with the C-N ratio of food eaten.** Average values (± standard deviation) are plotted for the four food sources tested (white square; D, DC, DS and nD), and for the food sources DS and DC, the feeding rate is additionally plotted in dependence of the body weight (dw) of the test organisms (+). The C-N ratio for the food source DC was also determined after classification into three groups of leaf colour (X).

Large variability in the C-N ratio of the food type DC was observed, and this was related to the colour of the leaf discs (Figure 4). The relatively large variability in the C-N ratio for the food type DC correlates with a large variability in feeding rate for gammarids fed with this food type. A further separation of the food source within one preparation procedure by nutrient content (here the C-N ratio) might reduce the variability of the test results even more than the 38.4% observed in this study, because the whole set of type DC leaf discs was used. Furthermore, it was observed that *G. pulex* had the smallest C-N ratio tested (5.55 ± 0.02) followed by *Cladosporium* sp. (10.32 ± 0.04). These C-N ratios are clearly smaller than those of all horse chestnut leaf discs tested (Figure 4). Benthic consumers often contain higher amounts of nitrogen and thus have a lower C-N ratio than their food sources [32]. There seem to be exceptions for this observation as the C-N ratio for the food source DS was close to that of the gammarids themselves (Figure 4).

Figure 4 also shows a relationship (*R*^2^ = 0.99) between the C-N ratio of food eaten (leaf discs excluding the veins) and feeding rate. The feeding rate decreased with increasing C-N ratio. The decline in the C-N ratio with decomposition time of the leaf discs is caused by microbial activity and was also observed for alder and beech leaves in the field [33]. This microbial activity, called conditioning, is an important part of leaf litter processing in aquatic ecosystems which increases the palatability of detritus for shredding organisms [8]. The literature suggests that aquatic shredders prefer food of lower C-N ratios because the quality is higher resulting in better nutritional status (original reference in [34]). However, whether food preference really depends on C-N ratio is unclear, as information in the literature range from no relationship between those two factors at all [34,35] to a strong relationship [36]. Nutritional composition is a determining factor of food quality [37], and adaptation in feeding activity provides a compensation for sub-optimal composition of available food [32,38].

Under the assumption that *G. pulex* only eats the amount of food needed to sustain the energy budget and nitrogen is the limiting factor, food consumption would increase as nitrogen content of the food decreased. However, the opposite relationship was observed (Figure 4). An explanation could be that the content of other important nutrients (e.g. phosphorus) might have been decreased during the decomposition by microbial activity and was then limiting. The compensation for this limitation then forced the gammarids to increase their feeding rate. For *G. fossarum*, another aquatic shredder, it has recently been shown that their growth, which is influenced by feeding, is negatively correlated to the C-P ratio of the food source [39]. Our results show that a comparison of feeding activity data generated in experiments using differing leaf species decomposed using different methods might be possible when the C-N ratio of the provided food is measured. However, it is advisable to include phosphorus into the testing of nutritional status of food sources; phosphorus is an essential component of food quality alongside carbon and nitrogen [37], and which nutrient is limiting depends on the actual content of the nutrient rather than the ratio.

### General discussion

Decreased intra-specific variability in feeding rate resulted when measurement of feeding rate focused on a sub-group of gammarids. Selecting organisms of a sub-group in terms of parasite infection and body mass resulted in a reduction in intra-specific variability of up to 50% and 57%, respectively. Using a food source of particular quality reduced the variability by up to 38%. Acclimation to test conditions only reduced the variability in test results by 1.6%. Certainly, taking into account each of these options to reduce the intra-specific variability for an ex situ feeding assay will maximise the reduction in intra-specific variability, but the result will not be additive.

There are contrasting strategies which can be followed for a feeding assay depending on the objective of the conducted study. The results suggest letting the organisms acclimate to the test conditions for at least 1 day and using organisms that are either all infected or all uninfected. The optimum should be the use of non-infected individuals as the infection tends to reduce feeding rate (present study and [21]), thus reducing the chance of measuring negative impacts due to the tested stressor. Furthermore, it is not known whether both parasites of *G. pulex* influence feeding in the same manner as here these parasites were not distinguished. The proportion of infection and the intensity of the infection with the parasites will depend on geographical location and season, and may in turn cause differences in impacts on feeding. Therefore, the use of uninfected organisms is recommended. Further research could include the investigation of xenobiotic impacts on feeding of infected organisms as those were shown to be more sensitive than uninfected organisms [21].

The results show that when conducting feeding assays with gammarids, attention has to be given to the selection of the test organisms in terms of their body size. In order to increase the chance of measuring influences of the treatment, the results suggest conducting experiments with organisms of a specific size class. Which size class to use might depend on the length of the planned study. Short-term experiments may be conducted with juvenile organisms of a very particular size because their feeding rate is in general higher than that for adults. Juveniles have been shown to have a higher sensitivity to toxicants [40,41], making this size class a good candidate for toxicity studies. A further reason to select juvenile individuals may be the increased representativeness of the test for the field situation. The density of organisms within the larger size class (adults) in the field is lower than that of smaller organisms [42]. However, as the feeding rate of juveniles fluctuates over time, they may only be suitable for short-term experiments. One may want to increase the number of replicates in such an experiment as the total amount of food consumed by juveniles within a day is rather low which increases the measuring uncertainty. Long-term experiments are particularly important to observe recovery potential following a treatment and for investigation of effects from pulsed exposure. Such experiments should be conducted with adult gammarids to stabilise the control feeding rate over time. A further reason to select adults is their importance for sustainability of the population as these individuals reproduce.

Some attention should be drawn to the food source to be used in a feeding assay. The results suggest using conditioned food prepared in a single batch and the C, N and P content of the food should be measured. Furthermore, more than one leaf disc should be provided per organism in order to reduce the variability of the feeding rate caused by the variability in the food quality. Generally, the longer the leaf material is inoculated with microorganisms, the higher is the feeding rate which, again, increases the chance of measuring negative impacts of the treatment. However, one might want to consider that there is likely a maximum feeding rate which is determined by the food handling time of gammarids. Conducting a feeding assay at such a shredding rate might eliminate the possibility to measure treatment-related increases (i.e. hermetic effects).

## Conclusion

There is no such thing as the perfect method which is valid for all research questions involving feeding assays with gammarids. The presented studies were not undertaken to develop such a method but aimed to enhance the design process to maximise the potential for successful and comparable experiments. We presented a list of factors that naturally have an impact on feeding rates of *Gammarus*, quantified their impact on the variability in feeding rates, discussed their importance for consideration when planning a feeding assay and suggested some additional measurements alongside the feeding assay to improve data comparison between studies.

## Methods

### Test organisms

We collected *G. pulex* from a small stream in Bishop Wilton, UK (grid reference: SE7963; latitude 53.985, longitude -0.787). The organisms were classified by visual observation immediately after collection into those infected (I) or uninfected (nI) with acanthocephalan parasites. The two parasites *Pomphorhynchus laevis* and *Polymorphous minutes* were not distinguished*.*

The organisms were maintained under continuous ventilation at 13°C ± 1°C and with a photoperiod of 12:12 h at 750 to 900 lux in artificial pond water (APW) (294 mg/L CaCl2 · 2H2O, 123.3 mg/L MgSO3 · 7H2O, 64.8 mg/L NaHCO_3_ and 5.8 mg/L KCl in deionised water) [13] prior to and throughout experimentation.

### Food sources

Food sources used did not represent the organism's preference of food. The food sources were chosen as a compromise combining the reported long-term survival of organisms when fed with this food, standardisation and thus reproducibility of food source production, and practicality of food source handling and storage. Overall we used five different food sources; four were prepared from horse chestnut leafs and one from alder leave. The horse chestnut leaves (*Aesculus hippocastanum* (L.)) were collected in November and stored after air drying under dry and dark conditions and at room temperature until use (20°C ± 2°C). Whole horse chestnut leaves were used as food to maintain the organisms in the laboratory (pre-experimental feeding). These leaves were stored in tap water at room temperature and were conditioned with *Cladosporium* sp. for at least 3 months prior to use. Horse chestnut leaf discs with a diameter of 1.6 cm were prepared for use in the experiments. Leaf types used differed in their decomposition state. These were decomposed by inoculation with *Cladosporium* sp. for 2 weeks (DC), decomposed with *Cladosporium* sp. for 3 months (D) and non-decomposed (nD). Culture media for the leaf type DC was enriched water (66.04 mg/L (NH_4_)_2_HPO_4_, 68.05 mg/L KH_2_PO_4_, 87.09 mg/L K_2_HPO_4_, 1.84 mg/L CaCl_2_ · 2H_2_O and 2.54 mg/L MgCl_2_ · 6H_2_O in deionised water) [13] and tap water for the leaf type D. Preparation of the leaf discs DC was by decomposing 150 horse chestnut leaf discs in 300 mL enriched water inoculated with *Cladosporium* sp. from a culture on malt extract. The leaf discs nD were prepared 2 days before the start of the experiment by storing them in tap water in the dark.

Alder (*Alnus glutinosa*) leaf discs (DS) prepared by inoculation with whole alder leaves previously inoculated in stream water were used as an additional food source. This food source was obtained from the University of Landau, Germany. Detailed information on the preparation of this food source can be found in Zubrod et al. [14]. In short, leaf discs with a diameter of 2.0 cm were conditioned for 10 days in a nutrient medium. Inoculation of the leaf discs with a river-like microbial community was by addition of alder leaves previously exposed in the Rodenbach, Germany (491330 N, 81020 E). The discs were dried at 60°C to constant weight and rewetted in APW 2 days prior to use in an experiment. This procedure was undertaken to guarantee a stable quality of the food sources (food quality within this study is defined as the C-N ratio of the food) over time.

### Experimental design

Artificial pond water was replaced every second or third day during all experiments; the oxygen content and pH in the old and new medium were measured. Mortality and moulting status were recorded daily. The feeding rate of moulting organisms was discounted from analysis during the period when the carapax was changed because the impact of moulting on the feeding rate is so far unknown; previous observations indicate that organisms might stop eating before moulting [43].

### Impact of food source and parasite infection

The first experiment consisted of five treatments, each with three replicates. Each replicate comprised four gammarids (body size 0.6 to 1.2 mm), six leaf discs at day one and three leaf discs during subsequent days in 250 mL APW. Three treatments contained uninfected gammarids and one of the three leaf discs D, DC or nD, respectively. Two treatments contained infected gammarids and either the food DC or D. The experiment lasted 96 h. Data for the first feeding period (*t*_0h_ to *t*_24h_) were excluded from further analysis because of a significant difference in feeding rate relative to subsequent periods. This experiment was conducted at the group level to minimise the variability due to size-dependent differences in the feeding rate.

### Measurement of C-N ratios

The carbon and nitrogen content of *G. pulex*, *Cladosporium* sp. and all leaf types was measured using a Vario MACRO CN elementar analyser (Elementar Analysensysteme GmbH, Hanau, Germany). Within the food type DC, three groups of leaves were analysed which were visually classified by their colour (light, middle and dark brown). The leaves and veins were analysed separately for calculating the C-N ratio of the leaf material consumed, because gammarids do not eat the main veins. Prior to analysis, the samples (duplicates) were dried (96 h at 105°C), milled and weighed. Sample weight ranged between 6.0 and 28.4 mg.

### Impact of body mass

Seventy-five organisms with a body mass between 0.48 and 14.6 mg dw were kept individually in 90 mL APW and fed daily with three leaf discs of the type DC. Only organisms without visible acanthocephalan parasite infection were used. The experimental period was 9 days.

### Individual feeding rate as a function of time for two food sources

Fifteen organisms with a body mass between 4.64 and 11.96 mg dw were kept individually in 90 mL APW. All organisms were fed daily with three leaf discs. Ten individuals were fed with the food source DC, and the remaining organisms were fed with leaf discs type DS. Only organisms without visible acanthocephalan parasite infection were used. The feeding rate of each individual was measured on a daily basis throughout the experimental period of 15 days.

### Measurement of feeding rate

To prevent over-estimation of the feeding rate associated with weight loss of the leaf discs caused by leaching and/or decomposition, the measured food at the end of the period *F*_(*t*)_ was corrected with a leaching-decomposition factor (ld). This factor was obtained by dividing the weight of the control leaves at the end of the measuring period by the initial weight. The actual amount of food eaten within the observed period *F*Ea_(*t*)_ (mg/d) (*F*Ea_(*t*)_ *= F*_(*t* − 1)_ − (*F*_(*t*)_/*ld*)) was then used to calculate the feeding rate *F*R (mg (food)/(mg (gammarid) × d)) at a daily resolution (*t* = exactly 24 h) by dividing the amount of food actually eaten within the observed period *F*Ea_(*t*)_ by the body mass of the individual *G* (mg) (*F*R *= F*Ea_(*t*)_/*G*). All measurements of weights refer to dry weight (dw).

Body mass of the organisms was measured after the experiment by drying the organisms for at least 24 h at 90°C. When the amount of food eaten was observed in wet weight (ww), the dw of the food material was calculated using the experimentally derived linear regression of dw = 0.186 × ww (*R*^2^ = 0.865; data not shown). Weighing was carried out with a Mettler Toledo XS205 Dual Range balance (Columbus, OH, USA) weighing to a precision of 0.01 mg.

### Statistical analysis

One- and two-way ANOVAs were performed with the feeding rate (mg (food)/(mg (gammarid) × d)) of replicates. The Shapiro-Wilk test for normal distribution and the Levene-Mediane test for equal variance were performed prior to ANOVAs. Multiple comparisons of resulting *p* values were by application of the Holm-Sidak test when normal distribution and equal variance were given. Otherwise, a Kruskal-Wallis test followed by an all pairwise comparison according to Dunn's method was used. Statistical analysis of feeding rates was undertaken with SigmaPlot 11 (Systat Software Inc., London, UK).
